# *In Vivo* Assessment of Genotoxic, Antigenotoxic and Anticarcinogenic Activities of *Solanum lycocarpum* Fruits Glycoalkaloidic Extract

**DOI:** 10.1371/journal.pone.0111999

**Published:** 2014-11-18

**Authors:** Carla Carolina Munari, Pollyanna Francielli de Oliveira, Luis Fernando Leandro, Leandra Mara Pimenta, Natália Helen Ferreira, Juliana de Carvalho da Costa, Jairo Kenupp Bastos, Denise Crispim Tavares

**Affiliations:** 1 Faculdade de Medicina de Botucatu, Universidade Estadual Paulista, Departamento de Patologia, Distrito Rubião Júnior, São Paulo, Brazil; 2 Universidade de Franca, Franca, São Paulo, Brazil; 3 Faculdade de Ciências Farmacêuticas de Ribeirão Preto, Universidade de São Paulo, Ribeirão Preto, São Paulo, Brazil; IIT Research Institute, United States of America

## Abstract

The fruits of *Solanum lycocarpum*, known as wolf-fruit, are used in folk medicine, and because of that we have evaluated both the genotoxic potential of its glycoalkaloidic extract (SL) and its influence on the genotoxicity induced by methyl methanesulfonate. Furthermore, the potential blocking effect of SL intake in the initial stage of colon carcinogenesis in Wistar rats was investigated in a short-term (4-week) bioassay using aberrant crypt foci (ACF) as biomarker. The genotoxic potential was evaluated using the Swiss mice peripheral blood micronucleus test. The animals were treated with different doses of SL (15, 30 and 60 mg/kg b.w.) for 14 days, and the peripheral blood samples were collected at 48 h, 7 days and 14 days after starting the treatment. For antigenotoxicity assessment, MMS was administered on the 14^th^ day, and after 24 h the harvesting of bone marrow and liver cells was performed, for the micronucleus and comet assays, respectively. In the ACF assay, male Wistar rats were given four subcutaneous injections of the carcinogen 1,2-dimethylhydrazine (DMH, 40 mg/kg b.w.), twice a week, during two weeks to induce ACF. The treatment with SL (15, 30 and 60 mg/kg b.w.) was given for four weeks during and after carcinogen treatment to investigate the potential beneficial effects of SL on DMH-induced ACF. The results demonstrated that SL was not genotoxic in the mouse micronucleus test. In animals treated with SL and MMS, the frequencies of micronucleus and extensions of DNA damage were significantly reduced in comparison with the animals receiving only MMS. Regarding the ACF assay, SL significantly reduced the frequency of ACF induced by DMH.

## Introduction

Genomic instabilities are induced by the usual metabolic processes, as well as by exogenous factors comprising diet, life style, and environmental stresses, such as solar radiation [1]. There is increasing evidence that cancer and other mutation-related diseases can be prevented not only by avoiding exposures to recognized risk factors, but also by favoring the intake of chemoprophylactic agents, as well as by modulating the defense mechanisms of the host organisms. This strategy, referred to as chemoprevention, can be pursued either by means of suitable pharmacological agents and/or by dietary factors [2]. Plants are considered among the main sources of biologically active chemicals, and a wide number of new compounds originated from natural resources have been developed into commercial drugs [3].

Plants of *Solanum* genus display anti-inflammatory [4], hypotensive [5], hepatoprotective [6], antiviral [7], antiurolithiasic [8] and antiallergic activities [9]. *Solanum* species are known for their high content of alkaloids, mainly for the two major glycoalkaloids, solamargine and solasonine, which are found in at least 100 species. Studies on the activities of these glycoalkaloids showed that it inhibit the growth of different cancer cell types, e.g. human colon (HT-29, HCT-15), human prostate (LnCaP, PC-3), human breast (T47D, MDA-MB-231, MCF-7), human hepatoma (HepG2, SMMC-7721), human cervical cancer (JTC-26, HeLa), human leukemia (K562), human glioblastoma (M059J, U343, U251), human osteosarcoma (U2OS), and murine melanoma (B16F10) [10]; [11]; [12].

*Solanum lycocarpum* A. St.-Hil. (Solanaceae) is a native plant found in the Southeast and West-Central Brazilian savanna called “cerrado”. The fruits of this plant, known as wolf-fruit, are used in folk medicine as antiepileptic, antispasmodic, hypoglycemic and hypocholesterolemic agent, as well as for controlling obesity [13].

Considering the frequent use of *S. lycocarpum* fruit in folk medicine and its medicinal properties, we have undertaken the genotoxic, antigenotoxic and anticarcinogenic activities of *S. lycocarpum* fruits glycoalkaloidic extract in rodents.

## Materials and Methods

### 1. Plant material, attainment and chromatographic analysis of alkaloidic extract

Fruits of *S. Lycocarpum* A. St.-Hil. Solanaceae were collected in the Campus of the University of São Paulo in Ribeirão Preto, São Paulo State, Brazil. The plant material was identified by Prof. Dr Milton Groppo Junior and a voucher specimen (code: SPFR: 11638) is deposited in the herbarium of the Faculty of Philosophy Sciences and Letters, University of São Paulo, Ribeirão Preto (SP), Brazil.

Fresh fruits were chopped and dried using an air circulating oven at 40°C. The alkaloidic extract was obtained by acid-base selective extraction [14]. The powdered dried fruits (1 kg) of *S. lycocarpum* were submitted to hydrochloric acid (0.5 M) extraction, three times sequentially, overnight by maceration, followed by percolation and centrifugation. Next, the aqueous acid extracts were alkalinized by adding NaOH (3.0 M) to achieve pH 12.0. Enough time was allowed for complete precipitation of the alkaloids. After precipitation, the supernatants were decanted and the wet precipitated biomass was centrifuged, combined and dried in an oven at 40°C. The dried rich alkaloidic extract was powdered in a knife mill, suspended in distilled ethanol and was submitted to sonication in an ultrasonic bath at room temperature (25°C). The ethanol soluble fraction was filtered, concentrated under reduced pressure and lyophilized to furnish the alkaloidic extract (9.2 g).

### 2. HPLC-DAD analysis

Chromatographic analyses were performed in an HPLC equipped with a diode array detector (HPLC-DAD) (*Shimadzu*) with Shimadzu Class-VP version 5.02 software, using a Zorbax SB-C18, 250×4.6 mm, colunm (*Agilent*). The glycoalkaloid analysis was undertaken employing an isocratic method of elution using acetonitrile/phosphate buffer (0.01 M) at pH 7.2 (36.5∶63.5). Standards of solasonine and solamargine were previously isolated from the alkaloidic extract. The flow rate was 1 mL min^−1^ for 20 min, and detection was set at 200 nm [14].

For the experiments, *S. lycocarpum* fruits glycoalkaloidic extract (SL) was dissolved in sterile distilled water.

### 3. Animals

Male Swiss mice (*Mus musculus*), 6–8 weeks old and weighing approximately 25 g, and male Wistar rats aged five weeks and weighing approximately 120 g were supplied by the Animal House of the School of Pharmaceutical Sciences, University of São Paulo, Ribeirão Preto (São Paulo, Brazil). The animals were kept in plastic boxes in an experimental room under controlled conditions of temperature (232°C) and humidity (5010%), under a 12-h light-dark cycle, with free access to regular laboratory diet chow and tap water. The study protocol was approved by the Ethics Committee for Animal Use of the University of Franca (process no. 033/009).

### 4. DNA damage-inducing agents

Methyl methanesulfonate (MMS, CAS: 66-27-3, Sigma-Aldrich, St. Louis, USA) was dissolved in distilled water and used as the positive control for the genotoxicity and antigenotoxicity tests. The MMS dose (40 mg/kgb.w.) was selected based on its effectiveness in inducing DNA damage.

The well-known colon carcinogen DMH (Sigma-Aldrich) was dissolved immediately before use in 1 mmol/L EDTA (ethylenediaminetetraacetic acid). A total dose of 160 mg/kg b.w. was divided into four subcutaneous (sc) injections of 40 mg/kg b.w., which was administered twice a week for two weeks (weeks 2 and 3).

### 5. Genotoxic and antigenotoxic assessment

The genotoxic potential was evaluated using the Swiss mice peripheral blood micronucleus test. The doses of SL were selected based on literature data [15] and preliminary experiments using the doses of 15, 30, 60, 120, 250, 500 and 1000 mg SL/kg b.w. The doses above 60 mg/kg b.w. showed to be toxic (data not shown). Therefore, the animals were treated by *gavage* (0.5 mL/animal) with 15, 30 and 60 mg/kg b.w. of SL or water (negative control) for 14 days. Peripheral blood samples were collected at 48 h, 7 days and 14 days after treatment for genotoxic evaluation. For antigenotoxicity experiments, on the 14^th^ day, it was administered MMS (i.p.; 0.3 mL/animal), and after 24 h it was performed the harvesting of bone marrow and liver cells, for the micronucleus and comet assays, respectively. Body weight and water consumption were measured throughout the experimental period.

### 6. Micronucleus assay

The in vivo peripheral blood micronucleus assay in bone marrow was performed according to the protocols described by MacGregor et al. [16]; [17]. For the determination of the frequency of micronucleated polychromatic erythrocytes (MNPCEs), 2000 polychromatic erythrocytes (PCEs) per animal were analyzed by light microscopy with a 100x immersion objective. A total of 400 erythrocytes per animal were scored to calculate the PCE/PCE+NCE (normochromatic erythrocytes) ratio in order determine the cytotoxicity of the treatments [18].

### 7. Comet assay

The alkaline comet assay was performed according to Burlisson et al. [19] under dim indirect light. Briefly, 20 µL of the liver cells suspension were mixed with 120 µL of molten 0.5% low-melting-point agarose and layered on a slide precoated with a thin layer of normal-melting-point agarose. The slides were placed into a lysis solution (2.5 mol/L NaCl, 100 mmol/L EDTA, 10 mmol/L Tris, 1% sodium laurylsarcosine, pH 10; with 1% Triton X-100 and 10% DMSO added just before use) for 24 h. Then, the slides were washed in PBS and placed into a horizontal electrophoresis unit filled with freshly made alkaline buffer (1 mmol/L EDTA and 300 mmol/L NaOH, pH 13). After 20-min of DNA unwinding period, electrophoresis was carried out in the same buffer at 25 V and 300 mA for 20 min. Afterwards, the slides were neutralized (0.4 mol/L Tris, pH 7.5) and fixed with 100% ethanol for 10 min.

All the slides of the experiment were coded before analysis. The slides were stained with 40 µL ethidium bromide solution (20 µg/mL in H_2_O) and covered with a coverslip. The comet DNA damage was visualized an AXIO Imager fluorescence microscope (Carl Zeiss, Germany) under 40× objective, and the images were captured with image analysis software (Comet imager V.2.0.0). DNA damage was quantified using a total of 100 nucleoids per repetition, resulting in a total of 600 nucleoids per treatment. The DNA damage was measured using DNA percentage in comet tail. As reported in an earlier paper [20], DNA percentage in tail is considered the best parameter for representation of DNA damage using comet assay.

For evaluating the viability, it was used the Trypan blue staining. Briefly, a fresh prepared solution of 20 µL Trypan blue (0.4%) in distilled water was mixed with 20 µL of each liver cell suspension obtained from comet assay, spread onto a microscope slide and covered with a coverslip. Non-viable cells appeared blue. At least 200 liver cells were counted per animal. Liver cells viability was calculated as a percentage of the respective control values.

### 8. Calculation of DNA damage reduction percentage

For antigenotoxicity experiments, the percentage reduction in MMS-induced damage by SL was calculated according to Waters et al. [21] using the following formula:
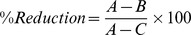
where *A* corresponds to the DNA damage observed for the treatment with MMS (positive control), *B* is the group treated with SL plus MMS, and *C* is the negative control.

### 9. Anticarcinogenic assessment

The anticarcinogenic potential of SL was performed by aberrant foci crypt (ACF) assay in Wistar rats. The preliminary experiments using the doses of 15, 30, 60 and 120 mg SL/kg b.w. in rats were evaluated. The doses above 60 mg/kg b.w. showed to be toxic, leading to weight loss and animal deaths. Each treatment group consisted of six animals fed with a regular chow throughout the experiment (five weeks). After one week of acclimation, animals were divided into six treatment groups, as following: SL (60 mg/kg b.w.); DMH (160 mg/kg b.w.); SL I (15 mg/kg b.w.) and DMH; SL II (30 mg/kg b.w.) and DMH; SL III (60 mg/kg b.w.) and DMH; and negative EDTA controls. Negative and positive control groups received EDTA (0.05 mL/10 g b.w.) and DMH (40 mg/kg b.w.), respectively, twice a week for two weeks (weeks 2 and 3). SL was administered to rats five times a week for four weeks by *gavage* (1.0 mL/animal), during and after DMH treatment. DMH and EDTA were administered by subcutaneous injections. All animals were anesthetized with sodium pentobarbital (45 mg/kg b.w., i.p.) and euthanized five weeks after starting the experiment (i.e., four weeks after the first DMH treatment). Body weight and water consumption were measured five times a week throughout the experimental period.

**Figure 1:**
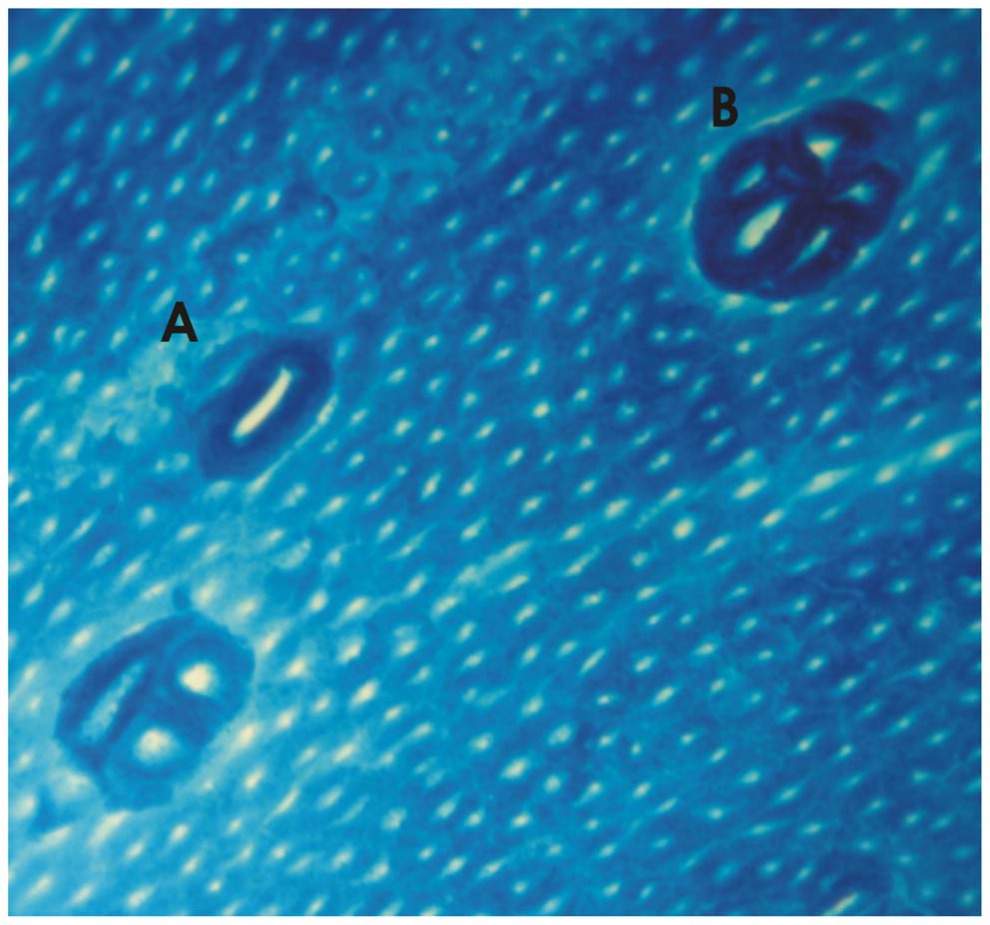
Photomicrograph of methylene blue-stained, whole-mount preparations of colon from Wistar rats treated with DMH with ACF contaning one (A) and five (B) aberrant crypts/focus.

After laparotomy, the colons were excised, flushed with 0.9% saline, cut open along the longitudinal axis, and fixed in 10% phosphate-buffered formalin (pH 6.9–7.1) for 24 h. Immediately before analysis, the colon was stained with 0.02% methylene blue for five minutes, mounted on microscope slides with the mucosal side facing upward, and observed under a light microscope at 100× magnification. Fifty sequential fields of the distal colon were screened for ACF, which were characterized by elongated, slit-shaped lumens surrounded by thickened epithelium that stained more intensely than the surrounding normal crypts [22] (Figure 1). The number of ACF and crypt multiplicity (number of crypts in each focus) was recorded. The multiplicity of ACF was expressed as aberrant crypts/focus. Each colon specimen was examined by at least three observers in a blinded manner.

### 10. Statistical analysis

The data were analyzed statistically by analysis of variance for completely randomized experiments (ANOVA), and a significant difference among treatment groups was evaluated by Tukey test. The results were considered statistically significant at p<0.05. All statistical analyses were made using GraphPad Prism 5.0.

## Results

### 1. Glycoalkaloids present in the *S. lycocarpum* alkaloidic extract

**Figure 2:**
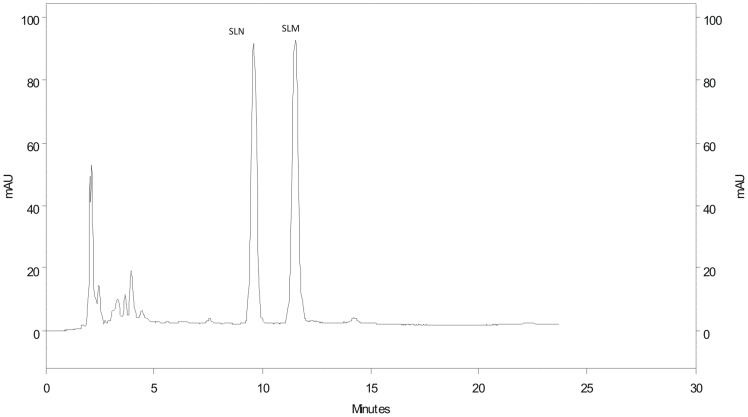
HPLC chromatogram of the alkaloidic extract of *Solanum lycocarpum* fruits. Zorbax-SB (5 µm), 250×4.6 mm column; mobile phase of acetonitrile and phosphate buffer 0.01 M (36.5∶63.5); flow rate of 1.0 mL/min and detection at 200 nm. SLN = Solasonine; SLM = Solamargine.

HPLC analysis of *S. lycocarpum* fruit’s glicoalkaloidic extract used in the present study, and obtained by selective extraction, allowed the quantification of two major glycolakaloids: solasonine (45%) and solamargine (44%) (Figure 2).

### 2. Genotoxic and antigenotoxic assessment

No significant difference in the frequencies of MNPCEs in peripheral blood was observed between animals treated with SL, for the three different period of sample collection (48 h, 7 days and 14 days), in comparison with the negative control (*P<*0.05). The frequencies of MNPCEs in bone marrow and DNA damage in liver cells demonstrated that the dose of 60 mg/kg b.w. was not statistically different from the negative control (*P<*0.05). These findings indicated the absence of genotoxic effects of SL, in the experimental conditions used in this study (Tables 1, 2 and 3).

**Table 1:** Frequencies of micronucleated polychromatic erytrocytes (MNPCEs) and polychromatic erythrocytes (PCE) to normochromatic erythrocytes (NCE) ratio obtained from peripheral blood of Swiss mice after treatment with SL and/or MMS, and their respective controls.

Treatments(n = 6/group)	48 h			7 days			14 days		
	MNPCEs		PCE/PCE+NCE	MNPCEs		PCE/PCE+NCE	MNPCEs		PCE/PCE+NCE
	N°	%	Mean ± SD	N°	%	Mean ± SD	N°	%	Mean ± SD
Control	38	0.31	0.09±0.04	33	0.28	0.09±0.04	45	0.38	0.07±0.02
SL 15 mg/kg	51	0.43	0.09±0.02	38	0.32	0.08±0.03	39	0.33	0.08±0.02
SL 30 mg/kg	41	0.34	0.10±0.03	31	0.26	0.07±0.02	34	0.28	0.08±0.02
SL 60 mg/kg	30	0.25	0.09±0.03	38	0.31	0.07±0.03	30	0.25	0.09±0.03
MMS	236^a^	1.97	0.09±0.03	–	–	–	–	–	–

MMS: methyl methanesulfonate (40 mg/kg b.w.); SL: *Solanum lycocarpum* fruits glicoalkaloid extract.

a Significantly different of the control group (*P*<0.05).

12000 polychromatic erythrocytes were analyzed per treatment.

**Table 2:** Frequencies of micronucleated polychromatic erytrocytes (MNPCEs) and polychromatic erythrocytes (PCE) to normochromatic erythrocytes (NCE) obtained from bone marrow of Swiss mice after treatment with SL and/or MMS, and their respective controls.

Treatments(n = 6/group)	PCE/PCE+NCE	MNPCEs		Reduction(%)
	MeanSD	N°	%	
Control	0.580.08	32	0.26	–
SL 60 mg/kg	0.590.04	23	0.19	–
MMS	0.580.08	281^a^	2.34	–
SL 15 mg/kg+MMS	0.580.04	176^a,^ b	1.46	42.17
SL 30 mg/kg+MMS	0.550.07	179^a,^ b	1.49	40.96
SL 60 mg/kg+MMS	0.560.06	171^a^^,^ b	1.43	44.18

MMS: methyl methanesulfonate (40 mg/kg b.w.); SL: *Solanum lycocarpum* fruits glicoalkaloid extract.

a Significantly different of the control group (*P*<0.05).

b Significantly different of the MMS group (*P*<0.05).

12000 polychromatic erythrocytes were analyzed per treatment.

**Table 3:** Mean percentage of DNA damage by the comet assay and cell viability by Trypan blue exclusion dye method in the liver cells of Swiss mice after treatment with SL and/or MMS, and their respectives controls.

Treatments(n = 6/group)	DNA in tail (%)	Reduction(%)	Cellviability (%)
	Mean±SD		Mean±SD
Control	13.56±3.61	–	100.00±0.00
SL 60 mg/kg	18.69±1.30	–	93.88±1.77
MMS	43.97±3.57^a^	–	98.35±1.25
SL 15 mg/kg+MMS	30.15±3.34^a^^,^ b	45.45	98.61±1.12
SL 30 mg/kg+MMS	29.64±3.74^a,^ b	47.12	97.75±1.30
SL 60 mg/kg+MMS	27.25±4.03^a^^,^ b	54.98	98.18±1.61

MMS: methyl methanesulfonate (40 mg/kg b.w.); SL: *Solanum lycocarpum* fruits glicoalkaloid extract.

a Significantly different of the control group (*P*<0.05).

b Significantly different of the MMS group (*P*<0.05).

600 nucleoids were analyzed per treatment.

The animals treated with different doses of SL associated with MMS showed significant reduction in both MNPCEs frequencies in bone marrow and DNA damage extensions in liver cells in comparison with the negative control. The reductions ranged from 40.96 to 44.18% in bone marrow cells and 45.45 to 54.98% in liver cells of Swiss mice. The gradual increase in the dose of SL did not result in a proportional increase in reducing the MMS-induced genotoxicity, indicating absence of a dose-response (Tables 2 and 3).

No significant reduction of PCEs in relation to the total number of erythrocytes was observed in any of the treatment groups in comparison with the negative control, evidencing the absence of cytotoxicity of the different treatments (Tables 1 and 1). The viability of liver cells by Trypan blue exclusion method was >93% for all experimental and control groups (Table 3).

No statistically significant differences in the body weight gain and water consumption, during the experimental period were observed between groups (Table 4).

**Table 4:** Initial body weight, final body weight, body weight gain and water consumption of Swiss mice after treatment for 14 days with SL and/or MMS, and their respective controls.

Treatments(n = 6/group)	Initial body weight(g)	Final body weight(g)	Body weight gain(g)	Water consuption(mL/animal/day)
Control	26.7±2.7	31.0±4.0	4.4±1.9	7.1±0.4
SL 60 mg/kg	26.9±1.7	30.0±1.5	3.1±3.2	6.5±0.7
MMS	29.6±1.2	34.1±3.0	4.6±3.2	7.5±1.4
SL 15 mg/kg+MMS	25.9±1.1	31.5±2.1	5.6±3.0	7.1±0.6
SL 30 mg/kg+MMS	26.8±1.6	29.5±1.8	2.8±2.5	5.9±0.6
SL 60 mg/kg+MMS	26.8±0.5	29.3±1.1	2.5±1.5	6.3±0.9

MMS: methyl methanesulfonate (40 mg/kg b.w.); SL: *Solanum lycocarpum* fruits glicoalkaloid extract.

### 3. Anticarcinogenic evaluation

Mean values and standard deviation of ACF and aberrant crypts observed in the distal colon of male Wistar rats are shown in Table 5. The groups of animals treated with EDTA (negative control) and SL (60 mg/kg b.w.) did not show ACF (data not shown). All DMH-treated groups developed ACF in the colon. In the 4-week ACF assay, the number of ACF and aberrant crypts were significantly reduced in the treated animals by 47%, 33% and 36%, respectively, for the doses of 15, 30 and 60 mg/kg b.w. of SL in comparison with the untreated group. Regarding the number of aberrant crypt/ACF, there was a prevalence of one aberrant crypt/ACF.

**Table 5:** Mean number (± SD) of aberrant crypt foci (ACF) and aberrant crypt in the distal colon of rats in response to SL during and after DMH treatment.

Treatments(n = 6/group)	Number of ACF with				Number of ACF	Number of aberrant crypt	aberrant crypt/ACF
	1 crypt	2 crypts	3 crypts	4 crypts			
DMH	8.33±1.37	2.17±0.75	0.33±0.55	0.0±0.0	10.83±1.72	13.66±2.58	1.26±0.09
SL 15 mg/kg+DMH	4.67±1.37	0.83±0.75	0.17±0.41	0.0±0.0	5.67±1.03^a^	6.83±1.17^a^	1.22±0.17
SL 30 mg/kg+DMH	5.67±1.51	1.50±0.55	0.0±0.0	0.0±0.0	7.17±1.47^a^	8.67±1.63^a^	1.22±0.08
SL 60 mg/kg+DMH	4.67±0.52	1.67±0.82	0.17±0.41	0.33±0.52	6.83±1.47^a^	9.33±3.14^a^	1.32±0.21

DMH :1,2 dimethylhydrazine (160 mg/kg b.w.); SL: *Solanum lycocarpum* fruits glicoalkaloid extract.

a Significantly different of the DMH group (*P*<0.05).

300 fields sequential/distal segment of the colon were analyzed per treatment.

Negative control animals and SL 60 mg/kg b.w. no showed ACF and aberrant crypt.

Treatment with SL resulted in a lower weight gain of the animals, although these differences were not statistically significant for the animals treated with the different doses of SL in comparison with the control group (Table 6).

**Table 6:** Initial body weight, final body weight, body weight gain and water consumption of rats after treatment for four weeks with SL and/or DMH, and their respective controls.

Treatments(n = 6/group)	Initial body weight(g)	Final body weight(g)	Body weight gain(g)	Water consuption(mL/animal/day)
Control	164.0±5.2	337.3±31.9	173.3±30.5	50.7±2.3
SL 60 mg/kg	171.7±6.6	312.0±21.6	140.3±23.0	43.0±1.7
DMH	166.5±7.4	369.0±22.5	202.5±23.5	51.1±1.4
SL 15 mg/kg+DMH	152.7±5.6	277.2±21.6	124.5±16.6	44.3±4.7
SL 30 mg/kg+DMH	156.3±13.0	280.7±38.5	124.3±33.6	37.8±1.1
SL 60 mg/kg+DMH	166.3±11.1	302.7±16.5	136.3±7.6	41.2±1.1

DMH :1,2 dimethylhydrazine (160 mg/kg b.w.); SL: *Solanum lycocarpum* fruits glicoalkaloid extract.

## Discussion

*S. lycocarpum* dried fruits bear approximately 1% of glycoalkaloids, and the use of standardized selective acid extraction allows to concentrate the glycoalkaloids in a precipitated fraction using only hydrochloric acid and water. To enrich the alkaloid content, it is necessary to submit the crude alkaloid fraction to digestion with ethanol. Then, it furnishes extracts with the content of glycoalkaloids ranging from 80 to 90% in a very reproducible way. Therefore, the developed extractions procedure is simple, reproducible and of low cost, which would allow the production of these alkaloids in an industrial scale.

The Swiss mice treated for 15 days with doses ≤60 mg/kg b.w. of SL showed no sign of toxicity, in the used protocols. However, SL led to lower weight gain to Wistar rats treated for four weeks. Previous studies showed that the administration of *S. lycocarpum* fruit did not produce body weight variations in male Wistar rats and Swiss mice, although a significant weight change was observed in some organs. Significant weight loss was observed only in the ventral prostate of mice receiving the high dose treatment [23]. Its administration to pregnant rats during the periods of blastocyst preimplantation and implantation [24] did not interfere with these processes. However, significant changes in the placenta, fetal, lung and kidneys weights were observed when administered to pregnant rats [25].

Maruo et al. [26] showed that long-term consumption of *S. lycocarpum* fruit can produce sight toxic effects in exposed adult rats. In this study, male (60 days treatment) and female (37 days treatment) adult rats received a diet containing 3% *S. lycocarpum* fruit. It was observed that while significant reductions in uterus and liver weight occurred in treated females, no alterations were reported in males. In addition, no significant differences were observed in other organ weights, blood enzymes or proteins.

Soares-Mota et al. [27] evaluated the toxic effects on male and female rats exposure to 10% *S. lycocarpum* fruit from weaning (21 days old) until adult age (8 weeks treatment). It was observed weight change in some organs of treated males, while in females, no differences were observed in organ weights. The histopathologic study showed no alterations between groups. Serum biochemical parameters showed triglyceride reductions in treated animals of both sexes. In females, an increase in both albumin and alanine aminotransferase levels, and a reduction in total protein levels were noted.

The present study demonstrated absence of genotoxicity of SL in Swiss mice micronucleus assay. The protective effect of SL against chromosomal and DNA damage induced by MMS were observed in Swiss mice bone marrow and liver cells, respectively. Furthermore, the SL significantly reduced the number of preneoplastic lesions induced by DMH, demonstrating anticarcinogenic activity. The chemopreventive effect can be attributed to the presence of the glicoalkaloids, solamargine and solasonine, major compounds in the glycolacaloidic extract of *S. lycocarpum*.

MMS has been used as an experimental model compound for several decades to elucidate the genotoxic mechanisms of alkylating agents. It is a direct monofunctional alkylating agent which monoadducts in the DNA, as well as crosslink causing mutations that involve different base substitutions [28]. There are reports of alkylating agents that rapidly replete gluthatione-S-transferase (GSH) in cells, thereby generating oxidative stress [29]. It has been postulated that the loss of GSH may compromise cellular oxidant defenses, with consequence accumulation of reactive oxygen species (ROS) generated as byproducts of normal cellular function. The generation of ROS may play a role in MMS-induced genotoxicity.

The DMH used to induce preneoplastic lesions is a carcinogen that requires metabolism within the organism to become active. The metabolic activation includes oxidation of DMH in azometane, azoximetane, N-hydroxylated and methylazoximetane. The methylazoximetane chemical structure is unstable at body temperature. During the process, it forms an oxidant agent, methyldiazonium, which generates a dioxide ion able to methylate the DNA, RNA and proteins [30]. DMH has been reported to induce carcinogenesis in rats and mice due to its high production of free radicals, which can result in cellular damage [31].

Although the mechanisms of action involved in chemoprevention of SL are not clear, some hypothesis can be drawn. The first is related to the reduction of the damage effects of ROS, due to possible indirect antioxidant action of natural product tested. *S. lycocarpum* fruits glycoalkaloid extract showed no genotoxic activity and revealed protective effect against both genomic and chromosomal damages induced by methy methanesulfonate [32]. Tavares et al. [15] demonstrated that the *S. lycocarpum* fruits hydroalcoholic extract not only exerted no genotoxic effect, but also it significantly reduced the frequency of chromosomal aberrations induced by DXR in both *in vitro* and *in vivo* mammalian cells. The authors attributed the chemopreventive effect observed to sum of interactions between different compounds of this complex mixture which contains glicoalkaloids and phenolic compounds, as major secondary metabolites. Both the retention times and UV spectra of the major peaks obtained in HPLC analysis confirmed the presence of solamargine (4.60%), solasonine (6.57%) and polyphenols (3.60%). It should be pointed out that the content of both alkaloids in the assayed alkaloidic extract was approximately 90%. Therefore, they might be mostly responsible for the reported activities.

Other species of *Solanum* have been investigated considering the mutagenic/antimutagenic potential. Chacon et al. [33] observed that the *S. melogena* hydroalcoholic extract had no mutagenic effect on Wistar rat bone marrow and peripheral blood cells using the micronucleus and chromosome aberrations assays. No mutagenicity was found in the immature fruit extract of *S. nigrum* in Ames test using TA 98 and TA100 strains, with or without the S9 mixture. However, *S. nigrum* extract showed antimutagenic effect for the same strains [34]. The presence of *S. nigrum* hydroalcoholic extract protected DNA against oxidative damage to its deoxyribose sugar moiety in the reaction mixture containing calf thymus DNA and free radicals generation system [35].

In plants of the genus *Solanum*, solasodine is a steroidal alkaloid and it occurs mainly as the glycoalkaloids solamargine and solasonine, which contain three sugar moieties, also called steroidal saponins. Hanausek et al. [36] observed 62% and 74% reduction by avicins, saponin (*Acacia victoriae*), in *H-ras* mutation at codon 61 during the initiation and promotion of skin carcinogenesis, respectively, as well as significant inhibition of the modified DNA base formation (8-OH-dG), inhibition of hydrogen peroxide generation, nuclear fator-κB (NF-κB) activation and nitric oxide synthase induction.

The second hypothesis is the modulating action of SL on the protein p53 and DNA repair genes. The protein p53, encoded by *TP53* gene, has been identified as a tumor suppressor protein to protect cells from DNA damage. Following cellular stresses, p53 is stabilized and binds to DNA as a tetramer, in a sequence-specific manner that results in the transcription regulation of genes that are involved in mediating key cellular processes, such as cell-cycle arrest, senescence, apoptosis and DNA repair [37]. There are evidences to suggest that the base excision repair (BER) induced by the base-damaging agent MMS is partially deficient in cells lacking functional p53. Base damage is frequently generated by ROS including superoxide, hydroxyl radical and hydrogen peroxide, and this DNA damage is corrected by BER. Moreover, base alkylation damages from endogenous alkylating agents (e.g., S-adenosylmethionine) and from monofunctional alkylating agents (e.g., MMS and vinyl chloride) are also repaired by BER [1]. According to Weissenberg [38] solasodine potential value as a precursor raw material for the synthesis of steroidal drugs is effective in the repair of DNA fragments.

In conclusion, under the present experimental conditions, SL did not shown genotoxic effects and revealed chemopreventive action. Additional studies involving the analysis of gene expression should be conducted to further elucidate the action mechanisms.
